# Comprehensive Analysis of Monocarboxylate Transporter 4 (MCT4) expression in breast cancer prognosis and immune infiltration via integrated bioinformatics analysis

**DOI:** 10.1080/21655979.2021.1951928

**Published:** 2021-07-16

**Authors:** Chen Yuan, Jie Zhang, Jianjuan Lou, Siqi Wang, Yanni Jiang, Feiyun Wu, Shouju Wang

**Affiliations:** Department of Radiology, The First Affiliated Hospital of Nanjing Medical University, Nanjing, Jiangsu, China

**Keywords:** Breast cancer, hexokinases-3, immune infiltration, monocarboxylate transporter 4, pyruvate kinase m2

## Abstract

Lactate blunts the anticancer immune response in breast cancer (BC). However, little is known about the exact effect of lactate transporters such as monocarboxylate transporter 4 (MCT4) on immunotherapy. In this study, we investigated the expression status and prognostic value of MCT4 in BC through large-scale transcriptome data. Our results showed that MCT4 was overexpressed in BC, particularly in the basal-like molecular subtype. Overexpression of MCT4 was significantly correlated with high BC lesion grade and poor prognosis. Enrichment analysis indicated that the MCT4-related genes were involved in immune- and metabolism-related bioprocesses, such as myeloid leukocyte activation, the adaptive immune system, and catabolic process. We also found that the expression of MCT4 in BC lesions was associated with immune cell infiltration and glycolytic rate-limiting enzymes like pyruvate kinase M2 (PKM2) and hexokinases-3 (HK3). Our observations indicate that MCT4 may play a pivotal role in the maintenance of the tumor immune microenvironment (TIME) through metabolic reprogramming. The enzymes of the glycolysis pathway (MCT4, PKM2, and HK3) may thus serve as new targets to modulate the TIME and enhance immunotherapy efficiency.

**Figure:**
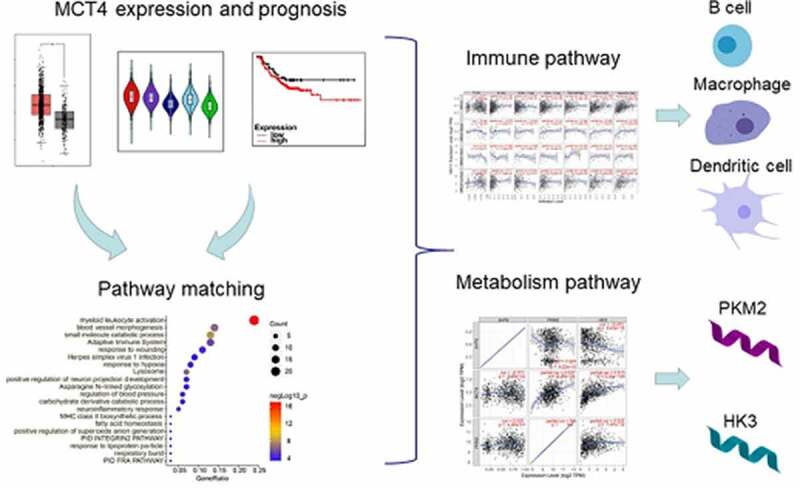

## 1. Introduction

Breast cancer (BC) is the most common malignant disease worldwide and estimated to account for approximately 30% of new cancer cases and 15% of cancer deaths[1]. In recent years, breakthroughs in the field of immunotherapy have provided new approaches for BC treatment [2,3]. Programmed death ligand 1 (PD-L1) [4] interacting with Programmed cell death 1 (PD1) to induce an immunosuppressive effect [5] has been the focus of immunotherapy. The Food and Drug Administration (FDA) has granted approval of PD-L1 inhibitor atezolizumab when combined with nanoparticle albumin-bound (nab)-paclitaxel for unresectable locally advanced or metastatic triple negative breast cancer (TNBC) [6], based on the results of the IMpassion130 trial [7,8]. However, some BC patients, even some TNBC patients with positive PD-L1 expression, are refractory to current immunotherapy [9]. These results indicate that the suppressive tumor immune microenvironment (TIME) in BC may not be completely reversed from the combination of PD-L1 inhibitor and chemotherapy. Thus, it is important to investigate the regulation mechanism of TIME in BC patients and to find new targets that will enhance the efficiency of immunotherapy.

Most BC cells exhibit distinct metabolic characteristics that promote their proliferation and progression, which is called metabolic reprogramming [10]. An altered metabolism has profound effects on the tumor microenvironment [11]. For example, high levels of glycolysis, regardless of oxygen concentration, result in increased lactate production and accumulation [12,13]. The tumor-produced lactate is internalized by cytotoxic T cells (CTLs) and inhibits their proliferation and anticancer function through the suppression of p38 and JNK/c-Jun activation, which is required for IFN-γ production [14]. The lactate can also polarize macrophages toward a tumor-promoting M2-phenotype and can exert the immunosuppressive function by activating G-protein-coupled receptor 132. Moreover, the anticancer immune response of dendritic cells (DCs) can be inhibited by lactate by inhibiting the differentiation of monocytes into dendritic cells [14]. Therefore, the elevated level of lactate is not only a by-product of BC glycolysis but also blunts the anticancer immune response in a concentration-dependent manner and plays a critical role in TIME modulation.

To maintain a high lactate level, monocarboxylate transporters (MCTs), particularly MCT1 and MCT4, make significant contributions [15]. The MCTs are encoded by the SLC16A gene family and function as a gate for the transport of lactate and other monocarboxylates [16,17]. However, only four proteins encoded by gene members of the SLC16A family are well studied and have known functions, namely, MCT1 (SLC16A1), MCT4 (SLC16A3), MCT2 (SLC16A7), and MCT3 (SLC16A8) [18,19]. Among MCTs, MCT1 and MCT4 are overexpressed in cancer and are related to poor prognosis [20–22]. However, the relationship among MCTs, TIME, and prognosis of BC patients is still unclear. Furthermore, some enzymes in the glycolysis pathway, such as pyruvate kinase and hexokinase, also regulate the production of lactate and might also exert an immune modulation effect.

To provide new insight into the potential function of MCTs and related enzymes in the TIME modulation of BC patients, the expression status and prognostic value of MCTs in BC were systematically investigated through large-scale transcriptome data involving more than 10,000 samples. In addition, the enrichment analysis to determine the MCT4-related bioprocesses was conducted. The association among MCT4, immune infiltration, and other glycolysis enzymes was also analyzed. This is the first comprehensive study to characterize MCT4 expression in BC patients from molecular, immune, and clinical aspects.

## 2. Materials and methods

### 2.1 Oncomine 4.5 database

The Oncomine 4.5 database (https://www.oncomine.org), with 715 datasets and 86,733 samples, is a comprehensive analysis platform for diverse cancers research from gene level, among which breast cancer accounts for the largest proportion containing 132 datasets with 14,277 samples in total [23]. Here, it served to discover the expression statues of MCT1 and MCT4 and to compare their expressive differences between normal tissues and cancers. The data of mRNA expression and DNA copy number of MCT1 and MCT4 in all types of cancers were investigated. ‘SLC16A1’ (MCT1) and ‘SLC16A3’ (MCT4) were input as key search words respectively with thresholds set as following: p = 1-E4, fold change = 2, gene rank = top 10%.

### 2.2 TIMER 2.0 database

The TIMER 2.0 database (https://cistrome.shinyapps.io/timer/) supplies multiple analysis methods from the immune aspect [24]. In this article, it was employed to assess the correlation of MCT4 expression with immune infiltration in different subtypes of BC. The immune cells included B cells, CD8 + T cells, CD4 + T cells, macrophages, neutrophils, and dendritic cells. It was also applied to question the relationship of MCT4, HK3 and PKM2.

### 2.3 Bc-genExMiner v4.5 database

bc-GenExMiner v4.5 database (http://bcgenex.centregauducheau.fr) is a systematic analysis tool for breast cancer transcriptomic data, including 10,716 DNA microarrays and 4712 RNA-seq [25]. In this study, the ‘expression’ module was applied to investigate gene expression profiles in different subtypes of BC using DNA microarray datasets (n = 10,001) and RNA-Seq datasets (n = 4712) respectively. Moreover, DNA microarrays data were explored in the ‘prognostic’ module to statistically identify the prognostic value of MCT4 for BC.

### 2.4 GOBO 1.0.3 database

The GOBO database (http://co.bmc.lu.se/gobo) is another analysis tool for breast cancer with 1881-samples [26]. It is designed in three different modules, data set module, web interface module and analysis module. Through the ‘Tumors’ selection in the analysis module, the expression levels of (SLC16A3) MCT4, hexokinase-3 (HK3) and pyruvate kinase M2 (PKM2), in all subtypes of BC were studied when they were input as gene symbols respectively. In this analysis, no multivariate parameters were (ER-status, Node status, Grade stratified, Age stratified, Size stratified) selected.

### 2.5 Kaplan-meier plotter database

The Kaplan-Meier plotter (http://kmplot.com) allows the acquirement of the effect of 70,632 genes on survival in 21 caners, especially for breast cancer (n = 6,234) [27]. Here, Kaplan-Meier survival curves supplied the access to investigate MCT4’s prognosis value for BC. In accordance with the lower quartile expression value, BC patients were split into high-MCT4 expression group and low-MCT4 expression group. Then the relationship of MCT4 expression and the relapse-free survival (RFS) (n = 3955) was obtained with the calculation of hazard ratio with 95% confidence intervals, and log-rank P value.

### 2.6 The metascape database

The Metascape Database (https://metascape.org) is a resource for gene annotation and analysis with its data updating monthly [28]. This tool was employed to study the biological process of MCT4 using the 100 most relevant genes of MCT4 in BC from the LinkedOmics database according to the Pearson correlation test. The genes were served for enrichment analysis in three modules (merged analysis, GO analysis, and KEGG analysis).

## 3. Results

As the overexpressed lactate exporter in BC, MCT4 is a potential target for modulating the TIME and improving immune therapy in BC patients. In this study, a comprehensive analysis was performed to investigate MCT4 expression signatures based on the largest transcriptome dataset available. The MCT4 level was associated with immune cell infiltration and prognosis of BC. In addition, the correlation between MCT4 and other main enzymes in the glycolysis pathway (PKM2, HK3) in BC were also characterized.

### 3.1 Expression signatures of MCT1 and MCT4

**Figure 1.**
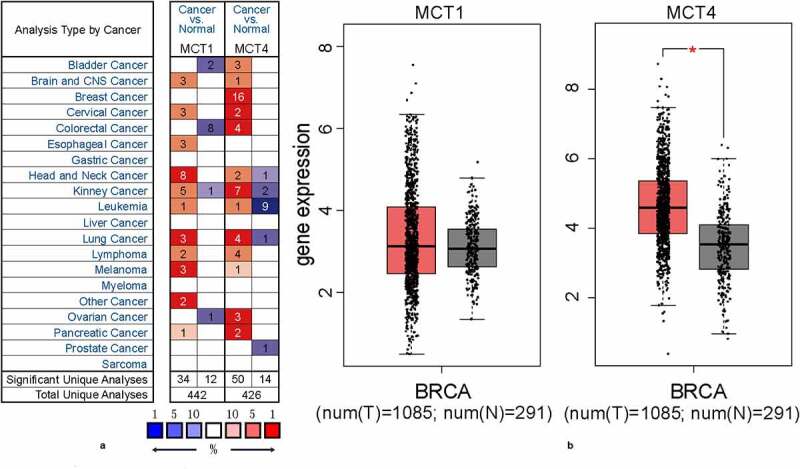
The expression profiles of MCT1 and MCT4 in tumor and normal tissues

First, the expression profiles of MCT1 and MCT4 in tumor and adjacent normal tissues were assessed using the Oncomine database (83 DNA datasets and 572 mRNA datasets, Figure 1a). MCT1 and MCT4 expressions were higher in most cancers. Remarkably, 16 analyses exhibited MCT4 overexpression in BC, while no analysis demonstrated upregulation of MCT1. Next, this study analyzed the difference in gene expression between BC and normal tissues based on The Cancer Genome Atlas (TCGA) and GTEx datasets. MCT4 exhibited significantly higher expression in BC than in normal tissues, while no marked difference was observed in MCT1 expression (Figure 1b). These results indicate that MCT4, not MCT1, has the potential to predict BC prognosis.

(A) The expression signatures of MCT4 and MCT1 in multiple cancers when compared with normal tissues. (B) The comparison of MCT1 and MCT4 expression levels in BC and normal tissues.

### 3.2 MCT4 expression profile in BC

**Figure 2.**
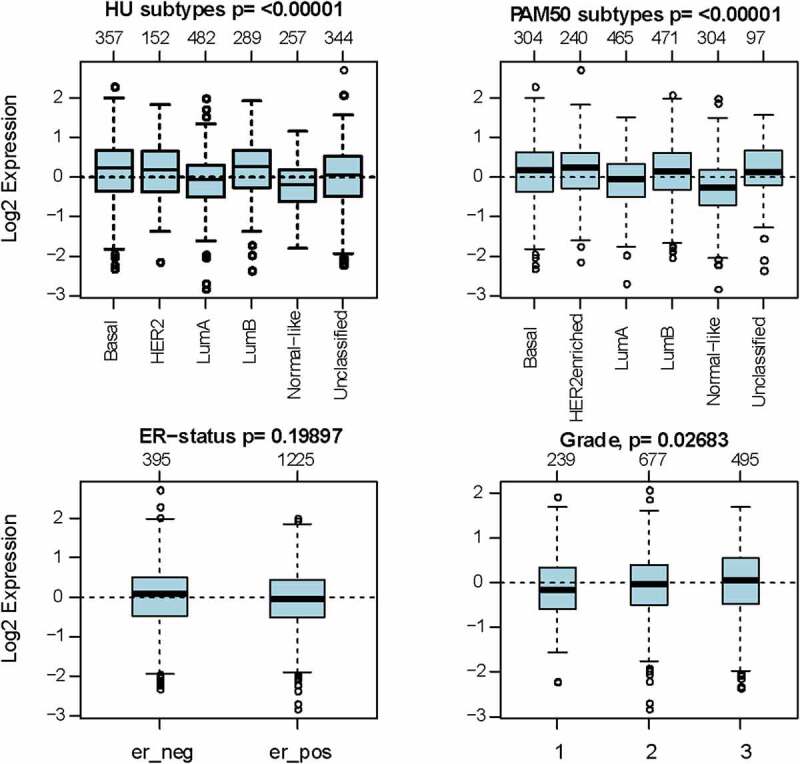
The expression pattern of MCT4 in different subtypes of BC using the GOBO dataset (n = 1881)

**Figure 3.**
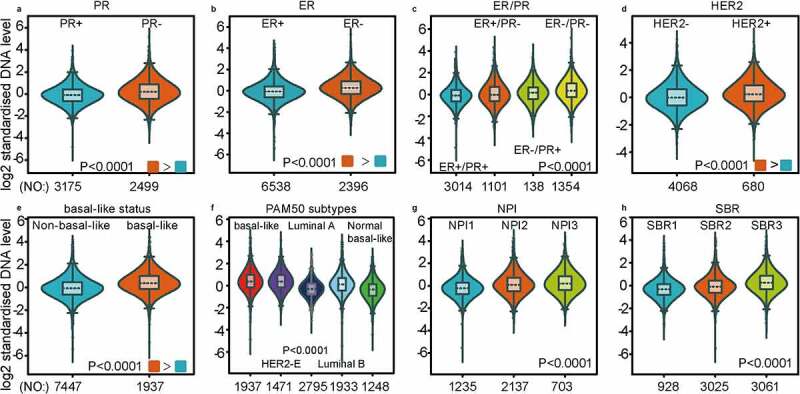
The expression profile of MCT4 in different subtypes of BC using the merged microarray datasets (n = 10,001)

**Figure 4.**
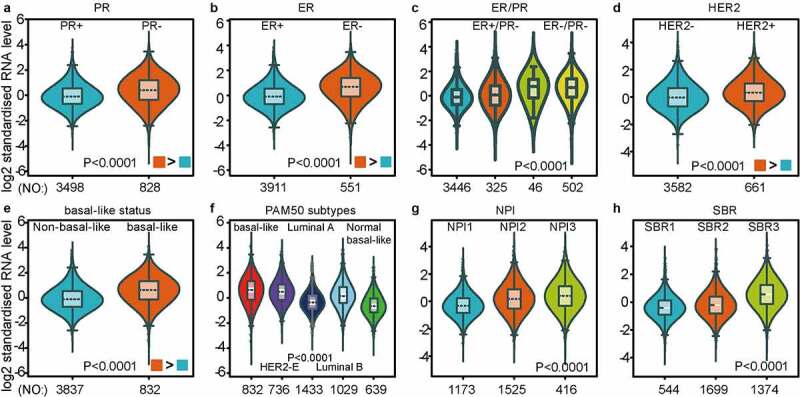
MCT4 expression signature from RNA-seq level exploiting merged RNA-seq datasets (n = 4712)

As investigated above, MCT4 was overexpressed in BC patients. Accordingly, the expression signature of MCT4 in BC was further explored using GOBO and bc-GenExMiner v4.5. First, GOBO (n = 1881) was applied to determine the expression levels of MCT4 in various clinical subtypes and stages (Figure 2). The overexpression of MCT4 was observed in basal-like, human epidermal growth factor receptor 2 (HER2)-enriched, and luminal B subtypes. Higher grade BC tended to have higher MCT4 expression. No association was observed between MCT4 expression and ER status. Then, bc-GenExMiner v4.5 (Figure 3) was used to further show the phenotype of MCT4. In the merged microarray datasets (n = 10,001), MCT4 expression was also higher in basal-like and luminal B subtypes. In addition, MCT4 expression was higher in progesterone receptor (PR)- and HER2+ groups than in PR+ and HER2- groups. Moreover, upregulation of MCT4 was associated with a higher Nottingham Prognostic Index (NPI) and Scarff-Bloom-Richardson (SBR) grade. However, the ER- BC group expressed higher levels of MCT4 than the ER+ BC group, which was inconsistent with the GOBO database analysis. The inconsistency perhaps resulted from the smaller amounts of samples in the GOBO database. Those are consistent with early reports suggesting that triple-negative breast cancer and high-grade disease were correlated with MCT4 expression [29,30]. Finally, the merged RNA-Seq datasets (n = 4712) were investigated to make the expression profile of MCT4 more credible (Figure 4). As expected, the results were in line with that of merged microarray datasets. Collectively, these results show that MCT4 was overexpressed in HER2-enriched and basal-like BC patients.

The expression profile of MCT4 is patterned by (A) PR state, (B) ER state, (C) ER & PR state, (D) HER2 state, (E) basal-like state, (F) PAM50 subtypes, (G) NPI grade, (H) SBR grade.NPI: Nottingham Prognostic Index (a clinicopathological tool for BC patients to stratify the prognostic risk); SBR: Scarff-Bloom-Richardson grade (histologic grading system for BC patients as a prognostic factor).

The expression profile of MCT4 in BC ranges from (A) PR state, (B) ER state, (C) ER & PR state, (D) HER2 state, (E) basal-like state, (F) PAM50 subtypes, (G) NPI grade, (H) SBR grade.NPI: Nottingham Prognostic Index (a clinicopathological tool for BC patients to stratify the prognostic risk); SBR: Scarff-Bloom-Richardson grade (histologic grading system for BC patients as a prognostic factor).

### 3.3 Relationship between MCT4 Expression and BC patient prognosis

**Figure 5.**
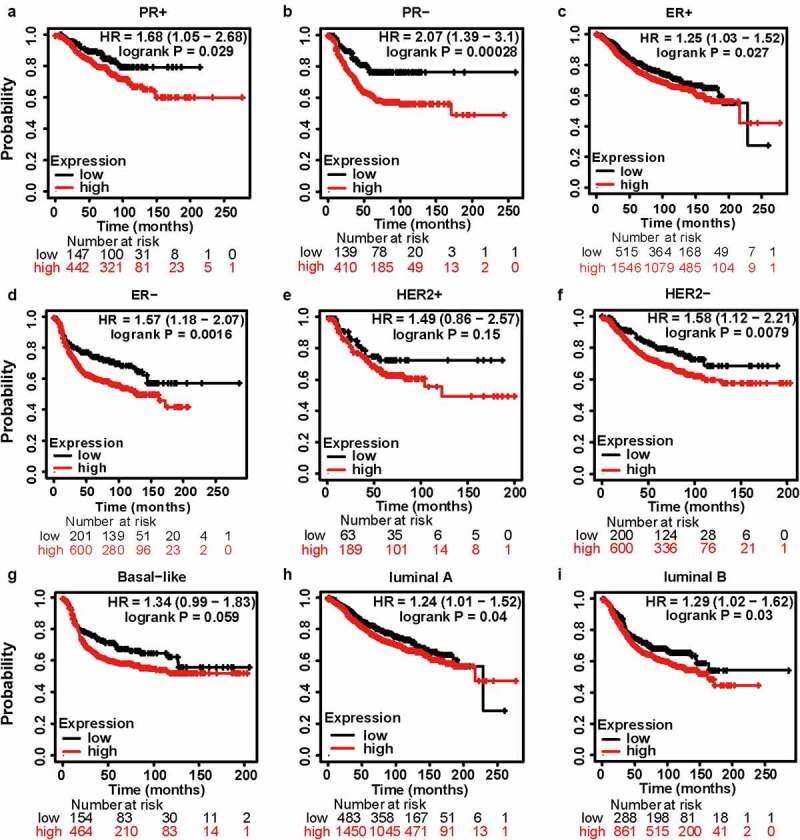
The kaplan-meier survival curves of high and low MCT4 expression in BC through the kaplan-meier plotter database. the RFS in (a) PR+ BC, (b) PR- BC, (c) ER+ BC, (d) ER- BC, (e) HER2+ BC, (f) HER2- BC, (g) basal-like subtype, (h) luminal A subtype, (i) luminal B subtype. RFS: relapse-free survival; HR: hazard ratio

**Figure 6.**
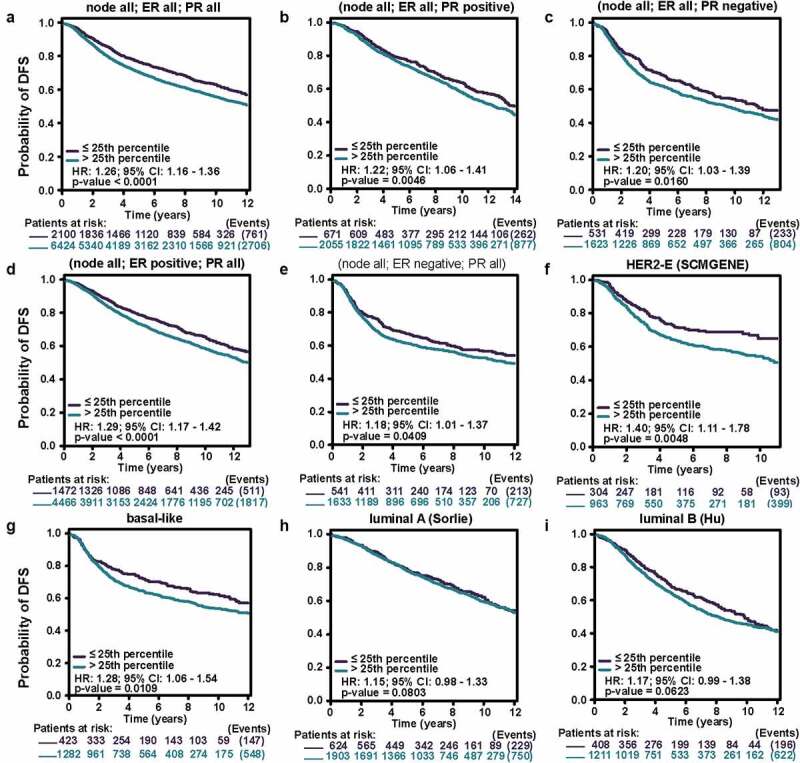
The kaplan-meier survival curves of high and low MCT4 expression in BC through the bc-GenExMiner v4.5

Encouraged by the results above, Kaplan–Meier analyses were conducted to explore the influence of MCT4 on patient prognosis. Among the immunophenotypes, higher MCT4 expression predicted worse relapse-free survival (RFS) (n = 3955) in PR+, PR-, ER+, ER-, and HER2- BC but not in HER2+ BC. Among intrinsic subtypes, MCT4 overexpression was associated with poor RFS in luminal A and B subtypes but not in basal-like subtypes (Figure 5). To validate the prognostic value of MCT4 in BC, this study further performed the prognostic analysis of MCT4 in the largest BC transcriptomic dataset, the bc-GenExMiner v4.5 database, using data from its DNA microarrays. Higher MCT4 expression was significantly accompanied by worse disease-free survival (DFS) (n = 8849) in BC, especially for PR+, PR-, ER+, and ER- BC. In contrast, among intrinsic subtypes, MCT4 overexpression predicted poor DFS in both HER2-enriched and basal-like subtypes (Figure 6). These results suggest that MCT4 is strongly related to the prognosis of HER2-enriched and basal-like subtypes in BC patients.

The DFS in (A) entire BC cohort, (B) PR+ BC, (C) PR- BC, (D) ER+ BC, (E) ER- BC, (F) HER2 enriched subtype, (G) basal-like subtype, (H) luminal A subtype, (I) luminal B subtype. DFS: disease free survival; 95% CI: 95% confidence interval.

### 3.4 Related biological processes of MCT4

**Figure 7.**
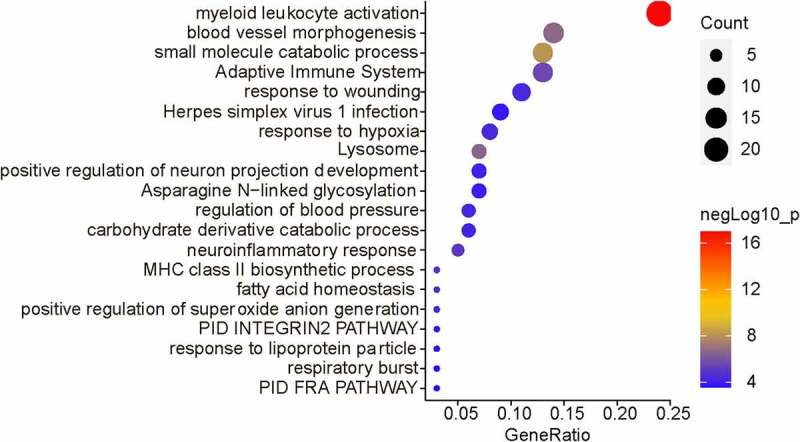
The biological functions of MCT4 showed in enrichment results

To further investigate the function of MCT4 in BC, the biological activities that involved MCT4 were explored. Through the LinkedOmics (http://www.linkedomics.org) database, the 100 most relevant genes of MCT4 in BC were screened from the TCGA database by Pearson’s correlation analysis (Pearson R > 0.45, Table S1). Then, enrichment analyses (Figure 7), Gene Ontology (GO), and Kyoto Encyclopedia of Genes and Genomes (KEGG) analysis (Figure S1) were conducted. The results indicate the MCT4-related genes participate in immune responses including myeloid leukocyte activation, adaptive immune system, and major histocompatibility complex (MHC) class II biosynthetic process, as well as metabolism-related bioprocess such as small molecule catabolic process, the carbohydrate derivative catabolic process, and fatty acid homeostasis. These results demonstrate that MCT4 affected not only the cancer metabolism but also the TIME.

### 3.5 Correlation between MCT4 expression and immune infiltrates in BC

**Figure 8.**
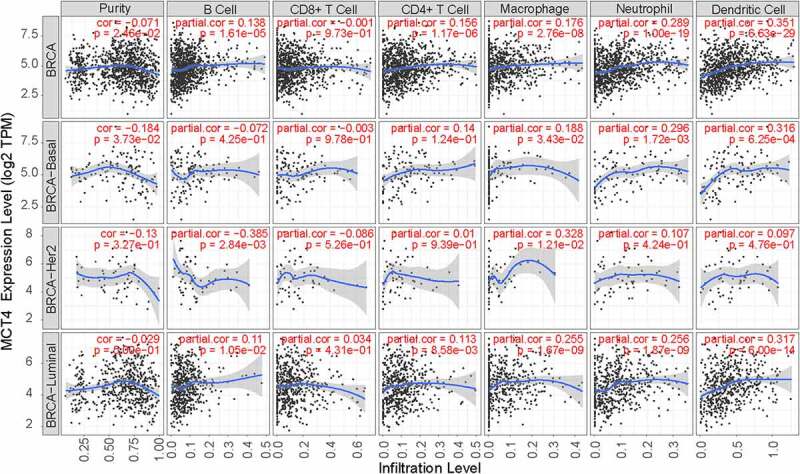
The relationship of MCT4 and immune infiltration in BC

The TIMER database was used to probe the correlation between immune infiltration and MCT4 expression in BC (Figure 8). The expression of MCT4 was positively correlated with dendritic cell infiltration in all BC patients (correlation = 0.351), especially in the basal-like subtype (correlation = 0.316) and luminal subtype (correlation = 0.317). Moreover, macrophage infiltration exhibited a positive correlation (the correlation coefficient was 0.328) with MCT4 expression in the HER2+ subtype while B-cell infiltration showed a negative correlation (the correlation coefficient was −0.385). These results imply that MCT4 expression in HER2-enriched and basal-like BC patients is related to immune infiltration, which is critical to the success of immune therapy.

### 3.6 Correlation between MCT4 Expression and carbon metabolism-related enzymes

**Figure 9.**
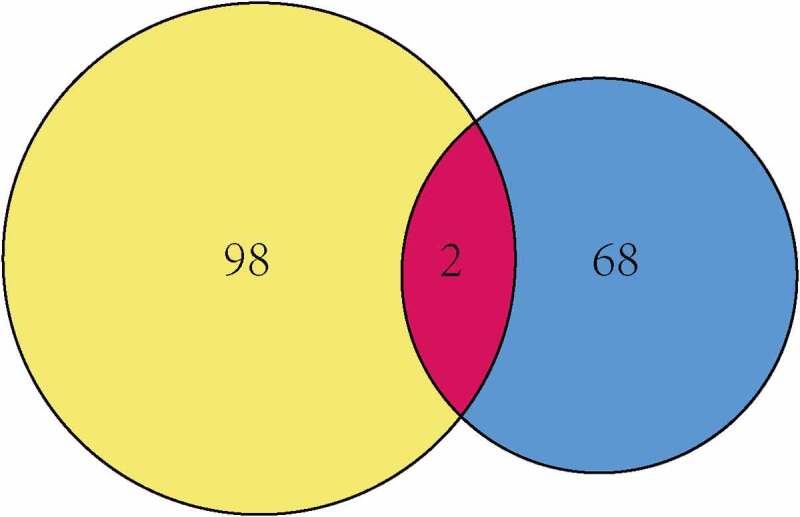
The intersection of MCT4 related genes and central carbon metabolism related genes

**Figure 10.**
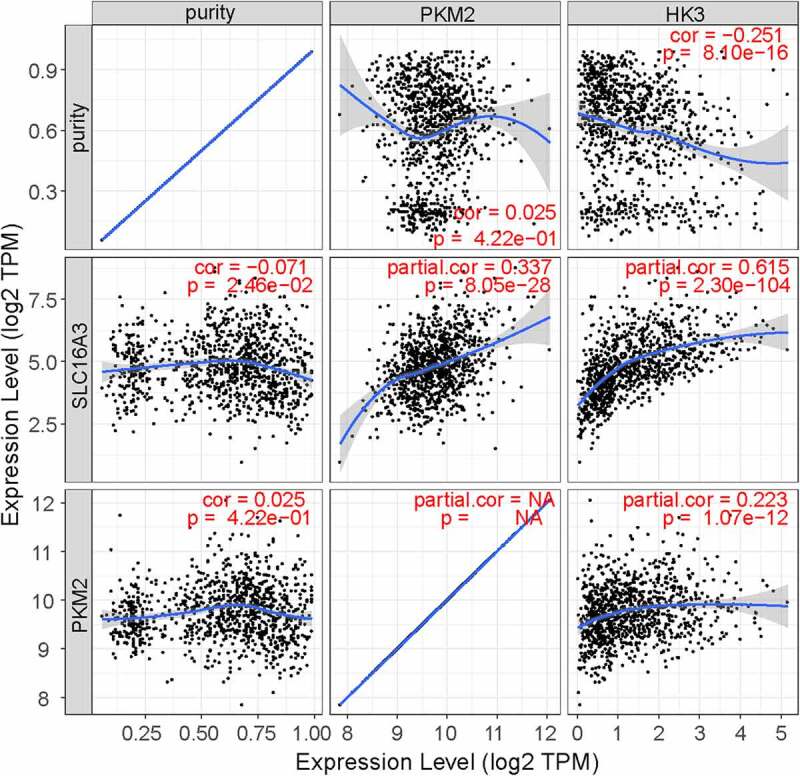
The relationship of MCT4, PKM2, and HK3

**Figure 11.**
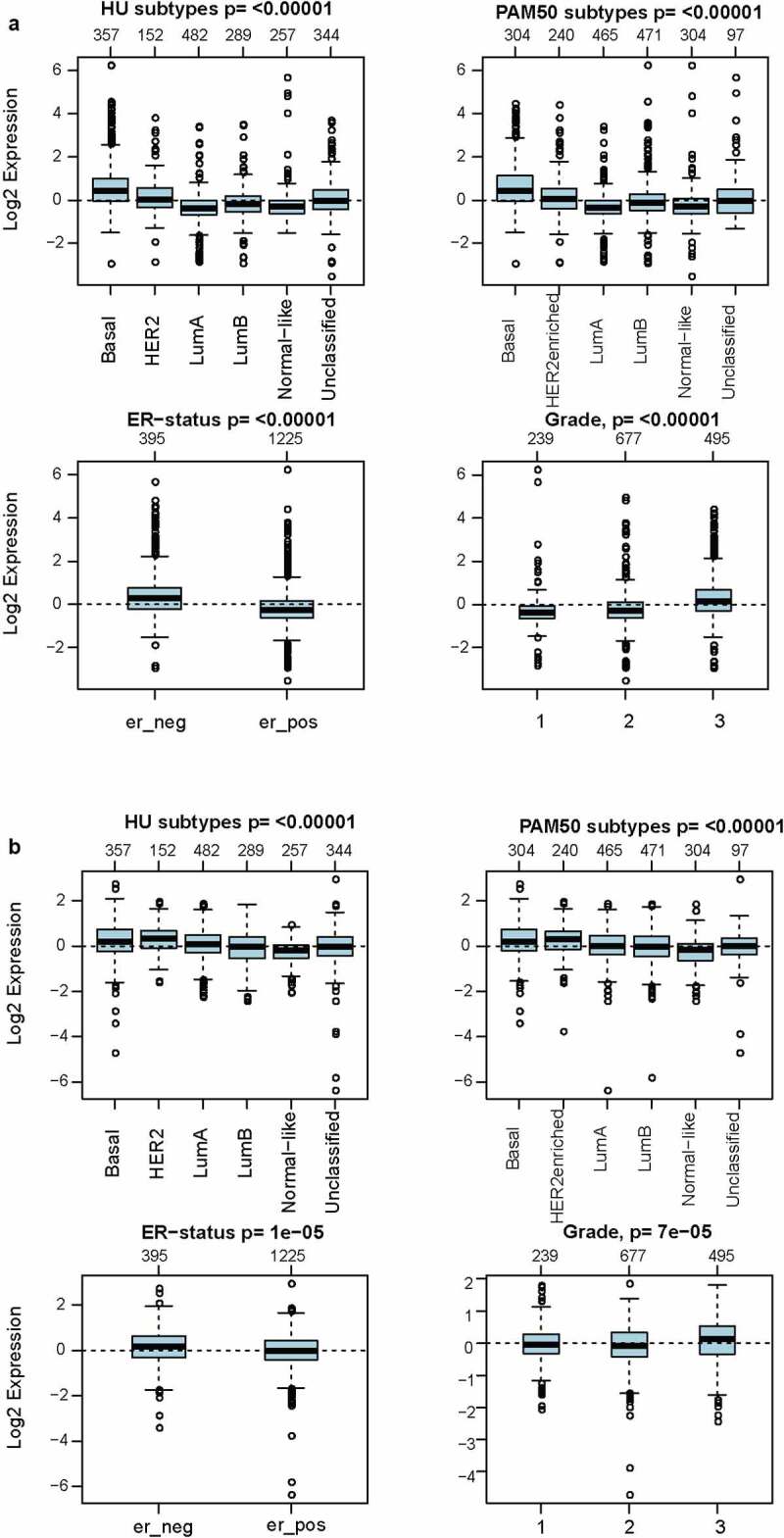
The expression signatures of (a) HK3 and (b) PKM2 in GOBO (n = 1,881)

Given that MCT4 engaged in metabolic reprogramming, the PathCards database was searched, and 70 genes in the central carbon metabolism pathway in cancer were obtained (Table S2). Interestingly, two enzymes, HK3 and PKM2, existed in both MCT4-associated genes and central carbon metabolism-related genes (Figure 9). Afterward, the study explored the relationship between MCT4, HK3 and PKM2 with tumor purity in the TIMER database. As exhibited in Figure 10, MCT4 was related to HK3 (partial.cor = 0.337) and PKM2 (partial.cor = 0.615), while HK3 and PKM2 did not show a significant correlation. The GOBO database was then applied to determine the expression statuses of HK3 and PKM2 (Figure 11). As expected, HK3 and PKM2 were both overexpressed in basal-like and HER2-enriched molecular groups, and ER- patients had higher HK3 and PKM2 expression than ER+ patients. Higher HK3 or PKM2 expression was also accompanied by a higher tumor grade.

## 4. Discussion

This study investigated the expression profile of MCT4 in BC, involving more than 10,000 samples and multiple verifications. These results showed that MCT4 was overexpressed in BC patients and was related to poor prognosis, particularly for HER2-enriched and basal-like subtypes. Moreover, MCT4 expression was related to immune infiltration in these two subtypes. Recent studies [31] have reported that higher levels of [13]C label exchange via MCT in more aggressive BC such as TNBC, which agrees with the observed MCT4 overexpression in basal-like subtype. Studies have also reported the potential of MCT4 as a new anticancer therapeutic target and prognostic indicator in other cancers, such as colorectal cancer [32] and prostate cancer [33]. Thus, it is anticipated that MCT4 will be a promising therapeutic target for BC, especially for TNBC. In fact, MCT4 has been demonstrated to be an important regulator of BC cell survival [29]. MCT4 inhibition caused intracellular lactate accumulation, increased reactive oxygen species levels, and induced cancer cell apoptosis [34,35].

The enrichment analysis found that MCT4-related genes were involved not only in metabolic but also in immune-related biological processes and pathways, such as myeloid leukocyte activation, the adaptive immune system, and catabolic process. MCT4 expression was significantly related to immune infiltration in BC. The TIMER database showed that MCT4 expression was related to dendritic cell infiltration in all BC patients (correlation = 0.351), especially in basal-like subtypes (correlation = 0.316). Macrophages and B-cell infiltration were correlated with MCT4 expression in HER2+ subtypes (the correlation coefficients were 0.328 and −0.385, respectively). These findings show moderate correlations among MCT4 expression, macrophages, B-cells, and dendritic cells. A recent study has reported that downregulation of MCT4 promotes the cytotoxicity of NK cells in BC [36], suggesting that MCT4 is involved in suppressive TIME. Another study has demonstrated that MCT4 is responsible for the immunosuppression effect attributed either to abrogating macrophage maturity or to disturbing T cell metabolism [37]. These results indicate that MCT4 modulates the TIME and has the potential to enhance the efficiency of immunotherapy in BC.

A significant correlation between MCT4 and rate-limiting enzymes in glycolysis (PKM2 and HK3, partial.cor = 0.337 and 0.615, respectively) was also observed. More importantly, these enzymes were also similar in expression and prognosis patterns as explored in the GOBO database. The results show that both HK3 and PKM2 are overexpressed in basal-like and HER2-enriched molecular groups, and ER- patients have higher HK3 and PKM2 expression than ER+ patients. Furthermore, higher HK3 or PKM2 expression was accompanied by a higher tumor grade.

PKM2 catalyzes the rate-limiting step of glycolysis and is explicitly expressed in various tumor cells [15]. The exact mechanism of PKM2 in regulating TIME is still under investigation [38]. A recent study has reported that TEPP-46, a well-characterized allosteric activator of PKM2, inhibits the pathogenicity of macrophages, DCs, T cells, and tumor cells [39,40], suggesting that inhibition of PKM2 has the potential to influence the TIME. As the first rate-limiting enzymes of glycolysis, HKs act in various cells (such as tumor and immune cells) [41]. Large pooled analyses were performed in HK2 [42], but few studies shed light on the HK3 mechanism. A previous study has showed that HK3 has a stronger relationship with tumor [17]F-FDG uptake than HK2, indicating its role in the activation of aerobic glycolysis [43]. Recently, researchers have also found that HK3 expression in tumor tissues may pertain to immune status [44], suggesting its capability to regulate the TIME via metabolic reprogramming, similar to MCT4 and PKM2.

There are several limitations in the study. First, all the data were retrieved from online databases, and the derived conclusion needs to be verified by external *in vitro/in vivo* experiments. Second, the prognosis value of MCT4 relative to overall survival of BC patients was not evaluated due to insufficient data. Finally, although the study showed MCT4, PKM2, and HK3 were associated with immune infiltration of BC patients, the specific mechanisms of how these enzymes modulate the TIME need further investigation.

## 5. Conclusion

In summary, MCT4 expression was observed to be associated with aggressive BC and poor prognosis. Our data also suggested that MCT4-related genes were involved in metabolic- and immune-related biological processes. MCT4 expression was consistent with immune cell infiltration in BC; therefore, MCT4 might be a promising target for anticancer application. Additionally, we propose that the main enzymes in the glycolysis pathway (MCT4, PKM2, and HK3) may function together by modulating TIME and enhancing the efficiency of anticancer immunotherapy in invasive BC, especially in HER2-enriched BC and TNBC.

## Supplementary Material

Supplemental Material

## 6. HIGHLIGHTS

- MCT4 was overexpressed in breast cancer, especially in the basal-like subtype.
- MCT4-related genes were involved in immune and metabolism related bioprocesses.
- MCT4 expression was associated with the immune cells and other glycolysis enzymes.
