# Supplementary material for: Comprehensive Analysis of Monocarboxylate Transporter 4 (MCT4) expression in breast cancer prognosis and immune infiltration via integrated bioinformatics analysis
Source: Bioengineered. 2021 Jul 16;12(1):3850–63. doi: 10.1080/21655979.2021.1951928 (PMC8806482; doi:10.1080/21655979.2021.1951928)
Supplement: Supplemental Material [file KBIE_A_1951928_SM5636.zip › supplementary.docx]

Supplementary Material


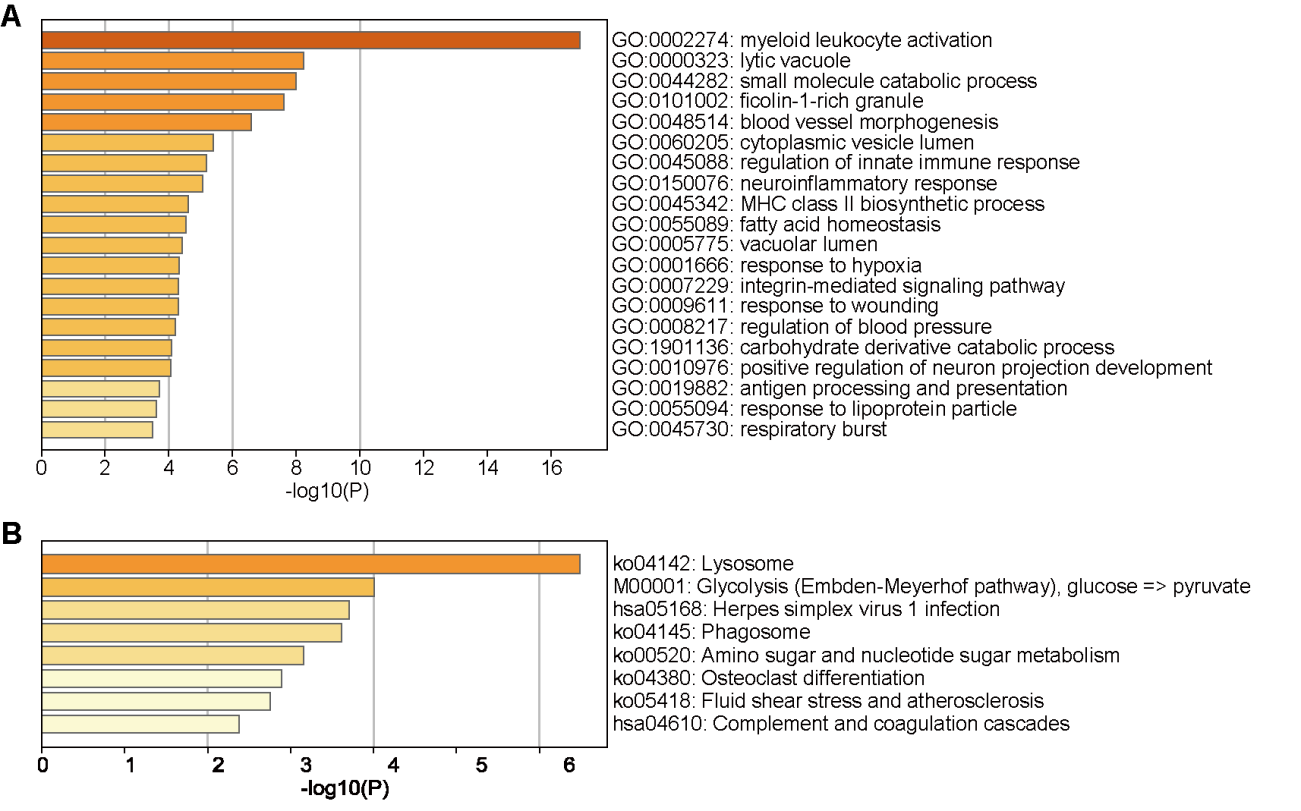


Figure. S1. The enriched results of MCT4 correlated genes

(A) GO pathway enrichment results of MCT4-correlated genes. (B) KEGG pathway enrichment results of MCT4-correlated genes.

Table S1. The 100 most relevant genes of MCT4 among all genes

| Gene | Person correlation | P-value |
| --- | --- | --- |
| HK3 | 0.600673884 | 3.76E-108 |
| ARPC1B | 0.592507167 | 1.46E-104 |
| IL4I1 | 0.583209736 | 1.35E-100 |
| SLC11A1 | 0.576299736 | 9.86E-98 |
| UPP1 | 0.568942437 | 9.32E-95 |
| PLAUR | 0.557261882 | 3.49E-90 |
| METRNL | 0.556263515 | 8.41E-90 |
| OSCAR | 0.551488387 | 5.45E-88 |
| CTSL1 | 0.550473562 | 1.31E-87 |
| LGALS1 | 0.544396512 | 2.36E-85 |
| TGFBI | 0.541182434 | 3.53E-84 |
| MMP9 | 0.53107777 | 1.44E-80 |
| TYMP | 0.530338501 | 2.62E-80 |
| CTSB | 0.527587336 | 2.38E-79 |
| ZFP14 | -0.524878591 | 2.06E-78 |
| TREM1 | 0.522205987 | 1.69E-77 |
| SLC2A6 | 0.520868613 | 4.83E-77 |
| TWF2 | 0.518365258 | 3.39E-76 |
| MAP7D1 | 0.514948244 | 4.73E-75 |
| ADRM1 | 0.512893671 | 2.27E-74 |
| IFI30 | 0.511711506 | 5.57E-74 |
| ADAM8 | 0.509725899 | 2.50E-73 |
| ABCD1 | 0.509708316 | 2.53E-73 |
| CCDC137 | 0.508015737 | 9.03E-73 |
| CTSZ | 0.507936101 | 9.59E-73 |
| C19orf28 | 0.507205812 | 1.66E-72 |
| GRN | 0.50265383 | 4.84E-71 |
| RPS6KA4 | 0.502176403 | 6.87E-71 |
| HBP1 | -0.501534939 | 1.10E-70 |
| SEMA7A | 0.500897583 | 1.75E-70 |
| GALK1 | 0.499738405 | 4.09E-70 |
| ZNF493 | -0.499596975 | 4.53E-70 |
| RGS19 | 0.498754742 | 8.36E-70 |
| ZNF561 | -0.4950358 | 1.23E-68 |
| FTL | 0.491014376 | 2.15E-67 |
| ZNF253 | -0.490623793 | 2.84E-67 |
| TMC6 | 0.489968089 | 4.51E-67 |
| CATSPER1 | 0.489830641 | 4.97E-67 |
| ZNF429 | -0.488598335 | 1.18E-66 |
| SCAI | -0.488246965 | 1.51E-66 |
| ZNF33A | -0.487713725 | 2.20E-66 |
| TNFRSF12A | 0.48759864 | 2.38E-66 |
| ZNF627 | -0.486230333 | 6.19E-66 |
| TMED9 | 0.486101009 | 6.78E-66 |
| CD300A | 0.484977309 | 1.48E-65 |
| ADM | 0.482969577 | 5.93E-65 |
| ZNF782 | -0.48269687 | 7.16E-65 |
| EMP3 | 0.481087361 | 2.16E-64 |
| ZNF181 | -0.480642449 | 2.93E-64 |
| FAM50A | 0.479460524 | 6.57E-64 |
| ARHGDIA | 0.478635054 | 1.15E-63 |
| MRPL12 | 0.478558076 | 1.21E-63 |
| SPI1 | 0.477220007 | 3.01E-63 |
| TMEM8A | 0.475625625 | 8.82E-63 |
| THOC4 | 0.475467358 | 9.81E-63 |
| CYBA | 0.475243678 | 1.14E-62 |
| METTL11A | 0.473946461 | 2.72E-62 |
| RELT | 0.473393192 | 3.94E-62 |
| PHLDA2 | 0.473348494 | 4.06E-62 |
| GPR84 | 0.471083601 | 1.83E-61 |
| RENBP | 0.470528236 | 2.64E-61 |
| CPEB3 | -0.470057465 | 3.61E-61 |
| SLC2A5 | 0.469886121 | 4.04E-61 |
| KCTD17 | 0.468619486 | 9.30E-61 |
| CD14 | 0.467923431 | 1.47E-60 |
| S100A4 | 0.467354682 | 2.13E-60 |
| SPHK1 | 0.467104516 | 2.51E-60 |
| RCN3 | 0.466779199 | 3.11E-60 |
| PLA2G15 | 0.466710552 | 3.25E-60 |
| HMOX1 | 0.466388592 | 4.01E-60 |
| ENO1 | 0.466146138 | 4.69E-60 |
| TYROBP | 0.465839377 | 5.73E-60 |
| RSBN1L | -0.465468584 | 7.30E-60 |
| APOE | 0.464755499 | 1.16E-59 |
| ZNF280D | -0.464151159 | 1.72E-59 |
| MGAT1 | 0.463841144 | 2.10E-59 |
| GLT25D1 | 0.463123534 | 3.33E-59 |
| MVD | 0.462671621 | 4.46E-59 |
| PSMA7 | 0.462637114 | 4.56E-59 |
| CTSA | 0.462187724 | 6.09E-59 |
| EMILIN2 | 0.462081918 | 6.52E-59 |
| ALDH6A1 | -0.461448039 | 9.80E-59 |
| CCM2 | 0.460875817 | 1.41E-58 |
| CD68 | 0.460167584 | 2.23E-58 |
| ATF7 | -0.460087745 | 2.34E-58 |
| PKM2 | 0.459962329 | 2.54E-58 |
| KIAA1737 | -0.459886027 | 2.66E-58 |
| FERMT3 | 0.459662053 | 3.07E-58 |
| UBN2 | -0.458952203 | 4.83E-58 |
| IKZF5 | -0.457821075 | 9.91E-58 |
| SIRT1 | -0.457310994 | 1.37E-57 |
| EFHD2 | 0.457273987 | 1.40E-57 |
| NARF | 0.45588221 | 3.38E-57 |
| RNF38 | -0.455759358 | 3.65E-57 |
| ANKRD58 | 0.455576828 | 4.09E-57 |
| KIFC3 | 0.45466085 | 7.28E-57 |
| TUBA1C | 0.454485009 | 8.13E-57 |
| C21orf70 | 0.45413736 | 1.01E-56 |
| ITGB2 | 0.454000039 | 1.10E-56 |

Table S2. Genes in the central carbon metabolism pathway in cancer

| AKT3 | SIRT3 | HRAS | MYC | PDK1 | PIK3R2 | SLC1A5 |
| --- | --- | --- | --- | --- | --- | --- |
| LDHAL6A | MTOR | IDH1 | NRAS | PFKL | PKM2 | SLC2A1 |
| EGFR | G6PD | IDH2 | NTRK1 | PFKM | MAPK1 | SLC2A2 |
| ERBB2 | GCK | KIT | NTRK3 | PFKP | MAPK3 | TP53 |
| AKT1 | GLS2 | KRAS | SIRT6 | PGAM1 | MAP2K1 | HKDC1 |
| AKT2 | GLS | LDHA | PDGFRA | PGAM2 | MAP2K2 | SLC7A5 |
| FGFR1 | HIF1A | LDHB | PDGFRB | PIK3CA | TIGAR | PIK3R3 |
| FGFR3 | HK1 | LDHC | PDHA1 | PIK3CB | PTEN | SLC16A3 |
| FGFR2 | HK2 | MET | PDHA2 | PIK3CD | RAF1 | LDHAL6B |
| FLT3 | HK3 | PGAM4 | PDHB | PIK3R1 | RET | SCO2 |
